# Panoramic/Dual-Surface Digital Image Correlation Measurement Using a Single Camera

**DOI:** 10.3390/s22093266

**Published:** 2022-04-24

**Authors:** Kaiyu Zhu, Bing Pan

**Affiliations:** Institute of Solid Mechanics, School of Aeronautic Science and Engineering, Beihang University, Beijing 100191, China; zkybh@buaa.edu.cn

**Keywords:** digital image correlation, panoramic measurement, experimental mechanics

## Abstract

We propose a cost-effective and simple-to-implement mirror-assisted single-camera panoramic digital image correlation (DIC) method for panoramic/dual-surface profile and deformation measurement. Specifically, two planar mirrors and a single camera attached with a four-mirror adapter are used to capture stereo images of the front and rear surfaces of a test object. These stereo images can be processed by regular stereo-DIC to retrieve shape and kinematics fields of each surface. Further, with the speckle patterns prefabricated on the mirrors, reflection transformation matrices are obtained and applied to transform all reconstructed surfaces into a common world coordinate system. As such, panoramic/dual-surface shape and deformation measurements can be realized. For validation, a high-resolution smartphone camera and an industrial camera were, respectively, used to construct mirror-assisted single-camera panoramic DIC systems. Real experiments, including panoramic shape measurement of an aluminum cylinder, dual-surface shape measurement of an aluminum plate and uniaxial tensile tests of aluminum sheet specimens, were performed, confirming the feasibility and accuracy of the method. Since only a single camera and a few auxiliary reflective mirrors are required, the proposed method provides a cost-effective and convenient way for taking panoramic/dual-surface shape and deformation measurements of regular-sized cylindrical and bar samples.

## 1. Introduction

Stereo-digital image correlation (stereo-DIC) is a practical and powerful non-contact optical technique capable of performing full-field profile and deformation measurement [1,2]. However, owing to ambient occlusion and limited field of view (FOV), regular stereo-DIC systems cannot measure 360-deg panoramic or dual-surface kinematics fields of a test object. For this reason, regular stereo-DIC cannot be applied for the direct or accurate determination of through-thickness strain, Lankford coefficient, true stress–strain curves and Young’s modulus of an eccentric tensioned sample, etc. [2,3,4,5,6], which necessitates the measurement of displacements and strains on multiple surfaces (panoramic or dual surface) of test objects.

To measure multiple-surface kinematics fields of a test object, various multi-camera DIC systems have been developed, as summarized in a recent review paper [7]. Specifically, in DIC community, multi-camera DIC system was first established by Orteu et al. in 2011 [8], known as the “Master-camera” configuration. The system comprised four synchronized cameras, one of which was appointed as the ‘‘master camera”, and the other cameras were, respectively, paired with it, forming three binocular stereo-DIC systems. However, on account of the requirements for common FOV between the master and subordinative cameras, the FOV of this multi-camera DIC system is very limited. Later, other multi-camera DIC systems with different configurations [7], such as “Camera-chain [9,10]”, “Connected-camera-pairs [11,12]”, “Face-to-face-camera-pairs [13,14]” and “Distributed-camera-pairs [15,16]”, were established. These multi-camera DIC systems either arrange all cameras into a chain and then pair any two contiguous cameras as a regular stereo-DIC system [9,10] or divide all cameras into a series of separated binocular stereo-DIC systems [11,12,13,14,15,16] to cover the full surface to be measured. As the requirements for the common FOVs are greatly reduced, these systems perform better in panoramic/multi-surface deformation measurements of objects with large sizes [17]. However, all these multi-camera stereo-DIC configurations comprise a series of real cameras, thus inherent shortcomings, such as high cost, complex system, large footprint and difficulty in camera synchronization, always exist, which hampers their applications in material testing of regular-sized samples.

Additionally, to achieve panoramic or large-scale measurement with a relatively simple and compact system, pseudo multi-camera DIC based on single or dual cameras have been proposed. For example, a binocular stereo-DIC system was moved to 16 positions to measure full-surface strain distributions of a nine-meter turbine blade [18]. By stitching two adjacent FOVs with reference points in overlapped fields, a continuous measurement over the entire surface can be obtained. Genovese et al. [19] proposed to achieve 360-deg full-surface measurement by rotating a single ordinary camera around the sample and the cylindrical calibration target. Alternatively, the camera was fixed while the sample was rotated [20]. In both methods, there is an overlapped FOV between any two adjacent locations, resulting in a series of regular binocular stereo-DIC measurements. Nevertheless, these pseudo multi-camera DIC systems necessitate moving either the camera(s) or the test object to capture the full object surface to be measured, which makes the experimental process complicated, time consuming and unsuitable for dynamic measurements [7].

Very recently, a novel mirror-assisted pseudo multi-camera DIC was proposed by Chen and Pan [7,17], which only requires one fixed conventional stereo-DIC system and two planar mirrors. With the calibration of reflection transformation, virtual surfaces reflected by the mirrors can be transformed to their actual positions, permitting 360-deg panoramic shape and deformation measurement. Compared with existing (pseudo) multi-camera DIC techniques, this method is simple in system configuration and easy to implement. The mirror-assisted pseudo multi-camera DIC has been successfully applied to determine many mechanical parameters, such as through-thickness strain [3], true stress–strain curve [6] and dynamic skin tension line [21]. Nevertheless, the use of two synchronized cameras still possesses the problems of relatively high cost in hardware investment and complexity in rigorous camera synchronization. These problems will be more prominent in the cases of transient deformation measurement employing dual high-speed cameras. Of course, a cost-effective and easy-to-implement single-camera panoramic DIC is of great benefit to research and educational efforts in resource-limited regions and institutes.

To this end, we propose a cost-effective and simple-to-implement mirror-assisted single-camera panoramic DIC for panoramic/dual-surface profile and deformation measurement. Compared with our recently proposed mirror-assisted pseudo multi-camera DIC, the main improvement of the proposed method is the replacement of the dual-camera stereovision system with a four-mirror-adapter-aided single-camera pseudo stereovision system. The use of single-camera pseudo stereovision system further reduces the cost and complexity in system construction and experimental implementation. To prove this concept, an easily available smartphone and an industrial camera, together with the corresponding auxiliary mirror adapters, were used to construct two different single-camera panoramic systems. Then, the established single-camera panoramic systems were used to achieve the 360-deg measurement or dual-surface measurement.

In the following sections, the system configuration and implementation procedures of the proposed single-camera panoramic DIC are first introduced. Then, the principle of reflection transformation and the validation experiments are detailed. Through panoramic shape reconstruction experiment of a regular cylinder, dual-surface shape reconstruction experiment of a plate and two uniaxial tensile tests of aluminum sheet specimens, the feasibility and accuracy of the proposed systems were verified.

## 2. Methods

### 2.1. System Configuration

**Figure 1:**
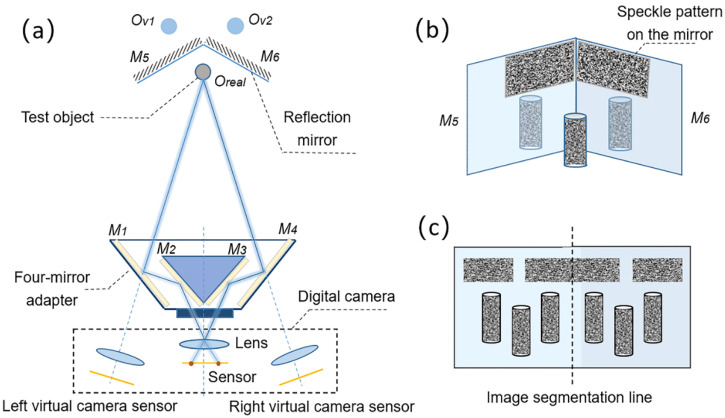
Schematic diagram showing: (**a**) optical paths of the proposed mirror-assisted single-camera panoramic DIC system, (**b**) the front view of the object and planar mirrors behind it, (**c**) the captured image.

As shown in Figure 1a, the proposed mirror-assisted single-camera panoramic DIC system consists of a digital camera, a four-mirror adapter and two planar mirrors placed behind the test object. During the measurement, with the help of the planar mirrors M_1_, M_2_, M_3_ and M_4_ in the adapter, two different views of the test object surface are, respectively, imaged onto the left and right halves of the camera sensor via different optical paths; thus, a binocular stereo vision can be formed with a single camera. The planar mirrors M_5_ and M_6_ are used to form two virtual images, O*_v1_* and O*_v2_*, of the real object O*_real_*, reflecting the part that cannot be observed into the observable area, as is shown in Figure 1b. A schematic diagram of the image captured by the system is shown in Figure 1c. Based on the reconstructed 3D shape of the speckles pre-prepared on the mirror surface with stereo-DIC, the shape and coordinates of the mirror surfaces can be acquired. With the equations of the planar mirror surfaces, the main reflection transformation parameters (distance to the origin and unit normal vector) [22,23] are obtained and applied to transform the virtual surfaces O*_v1_* and O*_v2_* to their real positions, achieving panoramic/dual-surface displacement and deformation measurement of a test object.

**Figure 2:**
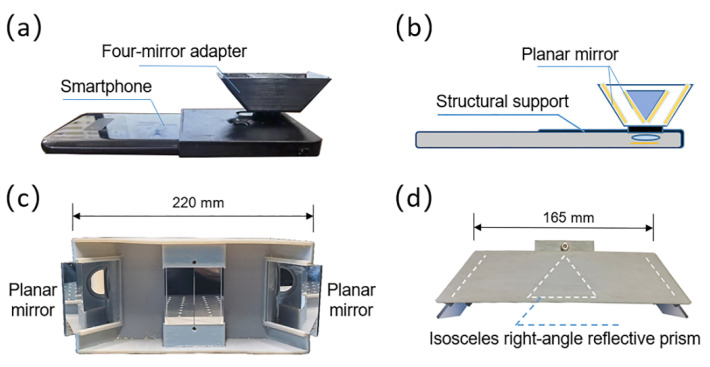
(**a**) The smartphone attached with a four-mirror adapter used in this work. (**b**) Cutaway view of the smartphone-based stereovision system. (**c**,**d**) are photographs of the front and top view of the four-mirror adapter in the industrial-camera-based stereovision system.

Here, we constructed two single-camera panoramic DIC systems with a smartphone and an industrial camera, respectively. For the smartphone-based stereo-DIC system, as shown in Figure 2a,b, the four-mirror adapter is attached in front of the smartphone. The two interior mirrors (M_2_ and M_3_, 28 mm × 21 mm × 1 mm) and two exterior mirrors (M_1_ and M_4_, 33 mm × 30 mm × 1 mm) are both glued on the 3D-printed structural support. The angle between M_2_ and M_3_ is 90°, while the angle between the exterior mirror and the baseline is 50°. For the industrial-camera-based stereo-DIC system, the four-mirror adapter is fixed on a tripod in front of the industrial camera. The two interior mirrors (M_2_ and M_3_) are formed by an isosceles right-angle reflective prism (height: 55 mm, hypotenuse: 40 mm), while two exterior mirrors (M_1_ and M_4_, 65 mm × 55 mm × 1 mm) are glued on the outside 3D-printed structural support. The angle between the exterior mirror M_1_ (M_4_) and the baseline of the virtual stereovision system is 53°, which is shown in Figure 2c,d (more details about the design of the four-mirror adapter can be seen in Ref. [24]).

Whether using ultra-portable smartphones or high-quality industrial cameras as image acquisition devices, the proposed method has the following three common advantages:

(1) Simple equipment and low cost. The equipment required in this system is just a smartphone (or an industrial camera) with a four-mirror adapter and two planar mirrors. Compared with conventional multi-camera DIC systems, the cost of panoramic/dual-surface measurement is greatly reduced.

(2) No requirement for the synchronization devices. This newly proposed panoramic DIC system guarantees the complete synchronization of all images without the need for synchronization devices, as images captured by two virtual cameras are in one picture for a single shot.

(3) Fewer experiment steps and easier implementation. As there is little equipment required, complicated wiring connections and the time-consuming equipment layout process are greatly reduced, especially when using a smartphone. Applying the proposed systems for a shape reconstruction experiment can generally be controlled within 20 min, which is unimaginable for a real multi-camera DIC system.

### 2.2. Measurement Procedures

**Figure 3:**
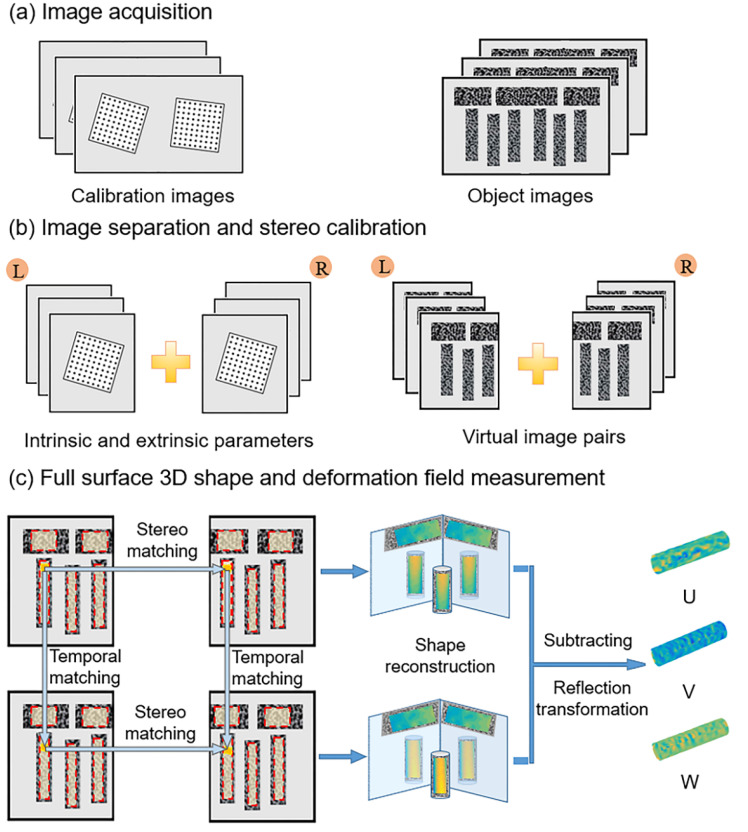
Implementation procedures of the proposed single-camera panoramic digital image correlation measurement.

As is schematically shown in Figure 3, the implementation procedures of the proposed single-camera panoramic/dual-surface DIC technique comprise three consecutive steps: (a) image acquisition, (b) image separation and stereo calibration, and (c) full-surface 3D shape and deformation measurement. Firstly, stereo images of the test object (with two planar mirrors behind it) and a regular calibration target are recorded by the single-camera panoramic DIC system. Then, the captured images are segmented into two parts, one of which only contains a single view of the virtual binocular system, along the segmentation line (i.e., the middle line). With the left and right calibration images, both the intrinsic and extrinsic parameters of the virtual binocular DIC system can be obtained. Through the stereo match of the image pairs, the disparity data used for the 3D profile reconstruction can be calculated.

The image pairs contain an image pair captured at the initial state and a series of image pairs recorded at deformed states after loading, which are designated as the reference images and deformed images, respectively. For each left or right image, five ROIs (regions of interest) are supposed to be designated (five for a cylinder, four for a plate.). The two ROIs covering the speckle patterns prefabricated on the M_5_ and M_6_ are merely employed to obtain the reflection transformation matrices, while the others are applied to retrieve the 3D profile, displacement and deformation fields of the test object. With these disparity data and calibration parameters, 3D coordinates and shapes of the ROIs at the initial state and deformed states can be retrieved and reconstructed based on the triangulation principle. Then, the reflection transformation matrices derived from the shape of planar mirrors are applied to the reconstructed virtual surfaces to obtain the panoramic/dual-surface profile of the test object at both the initial state and deformed states. Next, the full-surface displacement field of the object can be acquired by subtracting the 3D coordinates of the deformed state from those of the initial state. Finally, by differentiating the displacement field, panoramic/dual-surface deformation fields of the test object can be derived.

### 2.3. Estimation of Reflection Transformation of Mirrors

To acquire real coordinates of the virtual object surfaces, which is essential for the measurement of several important geometric and mechanical parameters, the reconstructed virtual surfaces are supposed to be transformed to their real positions with the reflection transformation matrix. As the principles of reflection transformation have been detailed in our previous work [4,17], here, only the reflection transformation estimation of the virtual surface reflected in M_5_ is briefly described.

**Figure 4:**
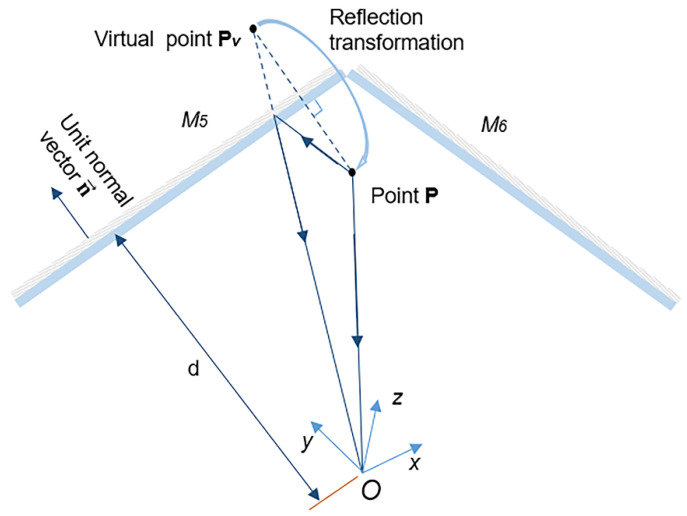
Schematic diagram showing the reflection transformation from virtual point **P***_v_* to its corresponding real point **P**.

As is shown in Figure 4, suppose the coordinates of an actual point **P** on the object are ${{(x}_{RP}{,\;y}_{RP}{,\;z}_{RP})}^{\;}$, the coordinates of the virtual point **P***_v_* reflected in the planar mirror M_5_ are ${{(x}_{VP}{,\;y}_{VP}{,\;z}_{VP})}^{\;}$, and the unit normal vector of the planar mirror M_5_ is $\vec{n}={{(x}_{M}{,\;y}_{M}{,\;z}_{M})\;}^{T}$. The distance from the origin *O* of the camera coordinate system to the planar mirror is *d*.

Then, we have

(1) $$\vec{OP}=\left[\begin{array}{c} x_{RP}\\ y_{RP}\\ z_{RP} \end{array}\right],\;\vec{OP_{v}}=\left[\begin{array}{c} x_{VP}\\ y_{VP}\\ z_{VP} \end{array}\right],\;\vec{n}=\left[\begin{array}{c} x_{M}\\ y_{M}\\ z_{M} \end{array}\right]$$

Through the reflection transformation matrix, the corresponding real point **P** can be obtained from the virtual point **P***_v_* as

(2) $$\left[\begin{array}{c} \vec{OP}\\ 1 \end{array}\right]=\left[\begin{array}{cc} \left(I_{3\times 3}-2\vec{n}{\vec{n}}^{T}\right) & 2d\vec{n}\\ 0_{1\times 3} & 1 \end{array}\right]\left[\begin{array}{c} \vec{OP_{v}}\\ 1 \end{array}\right]$$

The coordinate form of the equation is expressed as

(3) $$\left[\begin{array}{c} x_{RP}\\ y_{RP}\\ z_{RP}\\ 1 \end{array}\right]=\left[\begin{array}{cccc} 1-2x_{M}^{2} & -2x_{M}y_{M} & -2x_{M}z_{M} & 2dx_{M}\\ -2x_{M}y_{M} & 1-2y_{M}^{2} & -2y_{M}z_{M} & 2dy_{M}\\ -2x_{M}z_{M} & -2y_{M}z_{M} & 1-2z_{M}^{2} & 2dz_{M}\\ 0 & 0 & 0 & 1 \end{array}\right]\left[\begin{array}{c} x_{VP}\\ y_{VP}\\ z_{VP}\\ 1 \end{array}\right]$$

Using stereo-DIC, $P_{v}{=(x}_{VP}{,\;y}_{VP}{,\;z}_{VP}{)}^{T}$ and 3D profiles of the planar mirror M_5_ at both the initial and deformed states can be measured in a common world coordinate system. Therefore, with the plane fitting of the reconstructed shape of mirror M_5_, $\vec{n}={{(x}_{M}{,\;y}_{M}{,\;z}_{M})\;}^{T}$ and *d* in the reflection transformation matrix are all obtained, then the coordinates of the real point **P** are easily acquired.

## 3. Experiments

To examine the feasibility and measurement accuracy of the proposed single-camera panoramic DIC systems, four real experiments, including the panoramic shape reconstruction test of a cylinder object, dual-surface shape reconstruction test of a plate and two uniaxial tensile tests of aluminum sheet specimens were carried out.

### 3.1. 360-Deg Reconstruction Experiment

**Figure 5:**
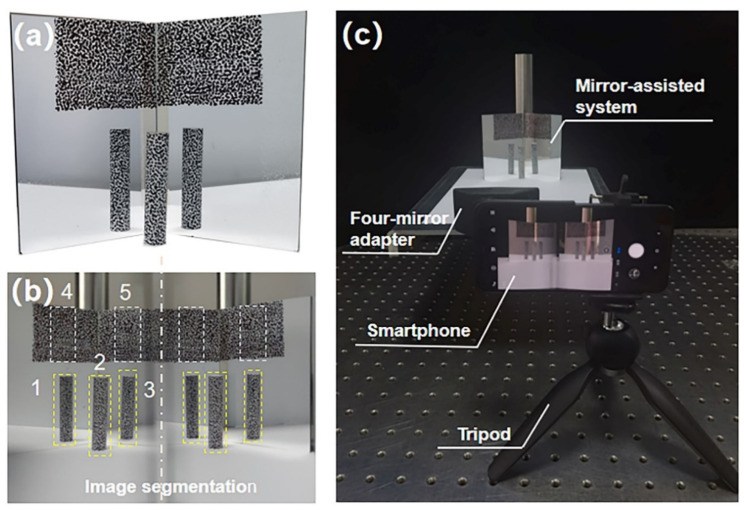
(**a**) Speckle pattern on the aluminum cylinder and two planar mirrors. (**b**) The image captured by the smartphone. The numbers indicated in Figure 5b denote five ROIs selected in DIC calculation. (**c**) The experiment setup of the 360-deg reconstruction experiment.

An aluminum cylinder (reference diameter: 9.98 mm) was first measured using the smartphone-based single-camera panoramic DIC system to verify the accuracy in 360-deg shape reconstruction. As shown in Figure 5a, before the experiment, a white background was first sprayed on the surface of the specimen with white matte paint; then, a 1 mm marker pen was used to create black speckle patterns on the background. Black speckles were also fabricated on the planar mirrors (M_5_, M_6_) with the same marker (without white background). The imaging system applied in this test is composed of a smartphone (Mi8, Android, camera sensor: Sony IMX363, 4032 × 3024 pixels, pixel size: 1.4 µm), a homemade optical attachment (as described in Section 2.1), two planar mirrors (100 mm × 70 mm × 1 mm) and a small tripod. Apparently, the smartphone accounts for the majority of the total cost of this portable panoramic DIC system, i.e., about USD 220. During the test, to avoid direct contact between the operator and the smartphone, a Bluetooth-based trigger was used to telecontrol the built-in capture software (manual mode). It is worth noting that, although the smartphone possesses two back cameras, only one (f/2.4) was applied for image capture. As shown in Figure 5c, the smartphone-based stereovision system was fixed on the tripod. The object distance was measured as about 260 mm. Two planar mirrors were placed behind the object and formed an angle of about 120 degrees.

**Figure 6:**
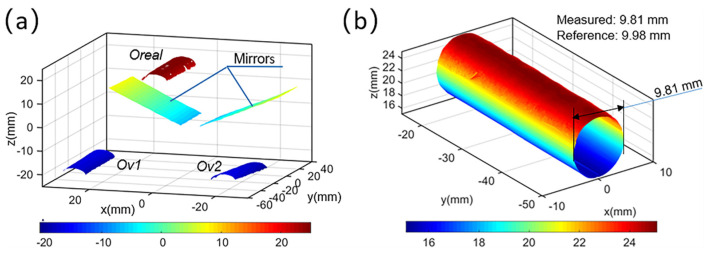
(**a**) Reconstructed shapes of the two planar mirrors and the cylinder object; (**b**) 360-deg full surface of the cylinder obtained with reflection transformation performed on virtual surfaces.

As is shown in Figure 5b, five surface pairs can be recorded by the single-camera stereovision system in a single shot, and five corresponding ROIs were selected on the left image. Based on the parameters of the smartphone system obtained by calibration, the shape of the three areas of the object and the two areas of the mirror are reconstructed with the subset-based DIC algorithm, which is shown in Figure 6a. The subset size and grid step for all ROIs in this experiment were designated as 41 × 41 pixels and 7 pixels. Then, reflection transformation is performed on the coordinates of the two virtual images of the objects, and the 360-deg panoramic shape of the cylinder shown in Figure 6b can be obtained. The fitted diameter is 9.81 mm, the relative error of which is less than 1.7% (reference size: 9.98 mm), proving the accuracy of the proposed single-camera panoramic DIC system for panoramic profile measurement.

### 3.2. Dual-Surface Reconstruction Experiment

**Figure 7:**
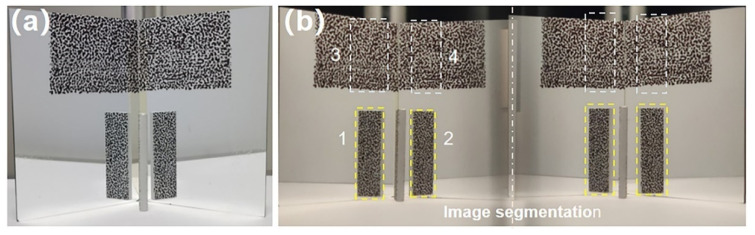
(**a**) Speckle pattern on the aluminum plate and two planar mirrors. (**b**) The image captured by the smartphone. The numbers indicated in (**b**) denote four ROIs selected in DIC calculation.

To further demonstrate the practicability of the proposed single-camera panoramic DIC system in panoramic/dual-surface profile reconstructions, we used the smartphone-based system to measure the dual-surface shape of an aluminum plate, the thickness of which was measured to be 4.10 mm using a Vernier caliper. Figure 7a shows the plate placed in front of the planar mirrors. The angle formed by the planar mirrors is about 105°. The image captured by the smartphone in the dual-surface construction experiment was shown in Figure 7b.

**Figure 8:**
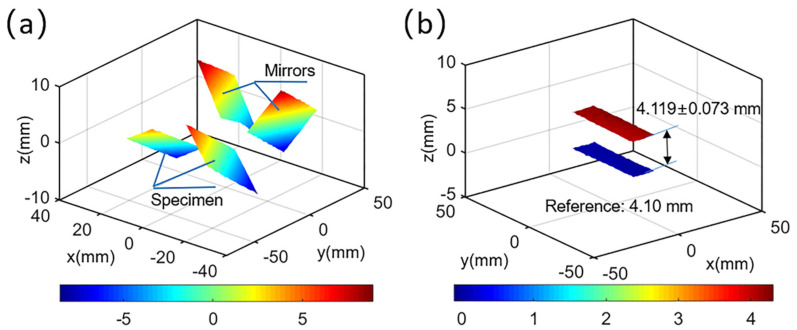
(**a**) Reconstructed shapes of the two planar mirrors and the plate; (**b**) dual surfaces of the plate object after reflection transformation.

As described in Section 2.2, the images captured were segmented into left and right parts along the middle line. Four ROIs were selected on the left reference image. Then, the subset size and grid step for the ROIs on both the test objects and mirrors were designated as 41 × 41 pixels and 7 pixels, respectively. With the calibration parameters of the imaging system and correlation results, 3D shapes of the test object (rear and front virtual surfaces reflected by mirrors) and two speckle regions on mirror surfaces were retrieved, which is shown in Figure 8a. After that, the reflection transformation matrices were estimated in accordance with the equations of the reconstructed mirror surfaces, and the real dual-surface shapes of the plate were finally retrieved. The results were shown in Figure 8b. The average thickness between the two surfaces was measured to be 4.12 ± 0.07 mm, and the error ratio was 0.487%, further proving the accuracy of the proposed single-camera panoramic digital image correlation system.

### 3.3. Uniaxial Tensile Experiment

To verify the feasibility and accuracy of the established single-camera panoramic DIC systems in real deformation measurement, two uniaxial tensile tests of aluminum sheet specimens (width: 10.0 mm, thickness: 1.6 mm, gauge length: 50 mm, reference elastic modulus: 68.2 GPa) were performed with a UTM.

**Figure 9:**
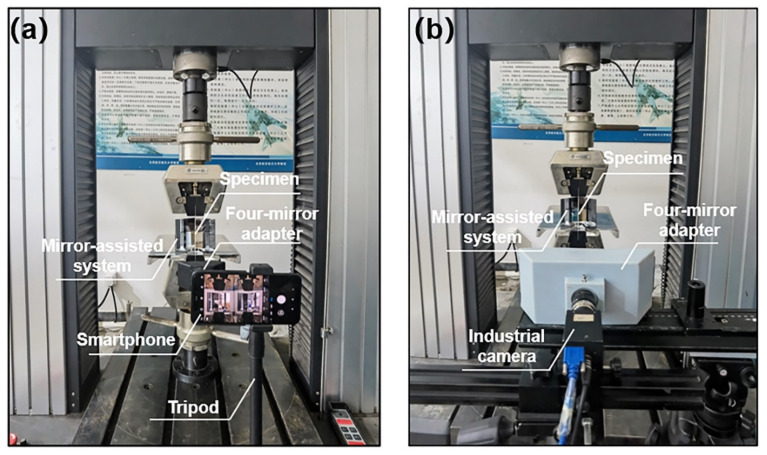
Experiment setup of the uniaxial tensile of aluminum sheet specimens using: (**a**) the smartphone-based system and (**b**) the industrial-camera-based system.

In the first experiment, to prove the dual-surface strain measurement accuracy of the smartphone-based single-camera panoramic DIC system, the smartphone system (the same as the one in the shape reconstruction experiment) was used to capture the deformation images, as is shown in Figure 9a. To eliminate the thermal-induced virtual strains caused by camera self-heating [25], the smartphone was preheated for 50 min by recording images continuously. Similarly, in the second experiment, to demonstrate the measurement accuracy of dual-surface strain of the industrial-camera-based single-camera panoramic DIC system, a high-resolution industrial camera (GS3-U3-91S6M-C, FLIR, 3376 × 2704 pixels; lens: Kowa, 25mm F1.4) and a four-mirror adapter (as is shown in Figure 2) fixed in front of the camera were used to capture the deformation images, as is shown in Figure 9b. The mirror-assisted system in both experiments is the same and fixed on a steel platform.

During the experiment, an image of the dual surface of the sheet specimen was first captured as the reference image. With a regular calibration target, the single-camera pseudo stereovision systems were carefully calibrated. In both experiments, the aluminum sheet specimen was loaded in increments of 100 N with the loading speed of 0.05 mm/min. A total of 23 images, including a reference image and 22 deformed images, were recorded and used for full-field displacement and strain calculation of the dual surface. It is noted that the maximum load applied was 2200 N and did not reach the elastic limit of the specimen. All the images were processed using the approach mentioned above.

#### Uniaxial Tensile Experiment Results

**Figure 10:**
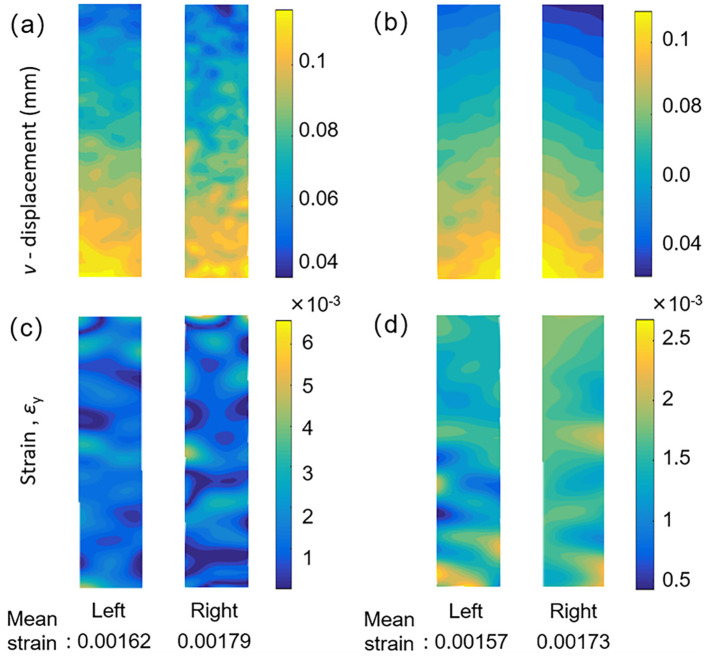
The longitudinal displacement (*v*) fields captured by: (**a**) the smartphone-based and (**b**) the high-resolution camera-based single-camera panoramic DIC system. (**c**) The longitudinal strain (*ε_y_*) fields captured by (**c**) the smartphone-based and (**d**) the high-resolution camera-based single-camera panoramic DIC system.

**Figure 11:**
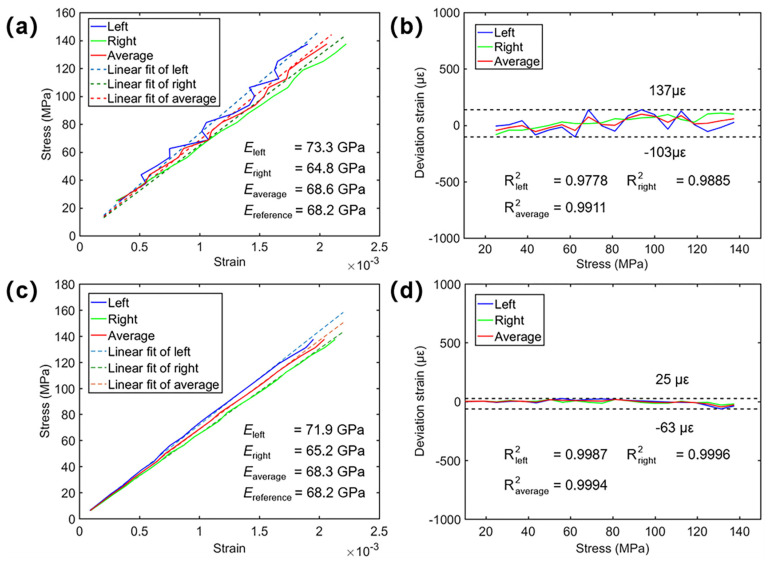
Tensile tests performed with the smartphone-based single-camera panoramic DIC system: (**a**) experimentally measured stress–strain curves and corresponding linear fitting (**b**) deviations between the measured and fitted strains. Tensile tests performed with the industrial-camera-based single-camera panoramic DIC system: (**c**) experimentally measured stress–strain curves and corresponding linear fitting (**d**) deviations between the measured and fitted strains. The R squared (R^2^) is the coefficient of determination, which provides a measure of how well the observed outcomes are replicated by the model.

Figure 10a shows the measured longitudinal displacements (*v*) and strains (*ε_y_*) on the front and rear surfaces with the loading force being 1800 N. In both experiments, the strain on the right surface is larger than the strain on the left because of the unavoidable eccentric loading in real tensile tests [4]. The measured dual-surface stress–strain curves of the specimen were plotted in Figure 11a,c based on the longitudinal strain and corresponding stresses at each state. Simultaneously, as suggested in Refs. [4,26], for homogeneous materials, by averaging the longitudinal strain (*ε_y_*) on dual surfaces of the specimen, elastic modulus can be determined with higher accuracy. As such, the averaged tensile stress–strain curves are plotted in Figure 11a,c as well.

The differences between the measured and fitted strains were calculated and plotted in Figure 11b,d. For the smartphone-based system, the standard deviations were estimated as 72 µε, 62 µε and 49 µε for the strain differences on the left, right and average. (µε denotes micro-strain, i.e., ε × 10^−6^). The deviation values are slightly larger than those of a professional stereo-DIC system. This is because the pseudo stereovision system constructed by the smartphone with a four-mirror adapter is more susceptible to the matching uncertainties and ambient interference than a professional system. Relatively shorter baseline distance and other differences in camera parameters may be the primary cause of this difference [25]. For comparison, the standard deviations of the strain differences measured by the high-resolution camera-based system were calculated as 20 µε, 11 µε and 14 µε for the surfaces on the left, right and average, verifying the robustness of the established single-camera system for dual-surface strain measurement.

The slopes of the three fitting lines were taken as the elastic moduli of the specimen (reference value: 68.2 GPa). For the smartphone experiment, the elastic moduli were estimated as 73.3 GPa and 64.8 GPa for the strain on the left and right surfaces. It is seen that a distinct deviation exists between the elastic moduli estimated from the strain of the two surfaces, while the one calculated from averaged stress–strain curve was 68.6 GPa and offers higher accuracy with a relative error of about 0.59%. For the high-resolution camera experiment, the obtained elastic moduli for the left, right surface and average strain are 71.9 GPa, 65.2 GPa and 68.3 GPa, respectively. The deviation between these two surfaces is also considerable, and the relative error of the elastic modulus calculated from the averaged stress–strain curve is only 0.15%. In both experiments, the elastic moduli obtained from the average of the dual-surface strains measured by the single-camera systems provided higher accuracy. These experimentally measured elastic moduli amply validated the ability of the proposed single-camera panoramic DIC system in dual-surface deformation measurement.

## 4. Conclusions

We propose a mirror-assisted single-camera panoramic DIC method for panoramic/dual-surface profile and deformation measurement of regular-sized test objects. Compared with the conventional panoramic DIC using two or more synchronized cameras, the proposed method offers distinct advantages of low cost, no need for synchronization, simple experimental steps and easy implementation. We established two single-camera panoramic DIC systems, applying a smartphone and an industrial camera as image acquisition devices, respectively. The feasibility and measurement accuracy of these two systems were verified by real experiments.

Finally, it should be noted that although the four-mirror adapter-assisted pseudo stereo-DIC is employed for stereo image capture in this work, other types of single-camera pseudo stereo-DIC systems [27], e.g., the X-cube prism-based full-frame single-camera stereo-DIC [28], can also be used. The proposed concept and established systems are expected to facilitate the application of panoramic DIC measurement in resource-limited laboratories and institutions.
